# Comparative performance of multiple novel obesity indices for MAFLD screening in Chinese children: the body roundness index as a practical predictor

**DOI:** 10.3389/fmed.2026.1773506

**Published:** 2026-03-26

**Authors:** Minhao Fan, Cai Tang, Shibo Lin, Jiahui Zhang, Xiaowei Zheng, Lihong Zhu, Le Zhang, Xiaomin Zhu, Yun Li

**Affiliations:** 1 Wuxi Key Laboratory of Genetic and Metabolic Diseases in Children, Department of Paediatrics, Wuxi Children's Hospital, Affiliated Children's Hospital of Jiangnan University, Wuxi, Jiangsu, China; 2 Department of Bariatric and Metabolic Surgery, The First Affiliated Hospital of Nanjing Medical University, Nanjing, China; 3 Department of Pediatric Laboratory, Wuxi Key Laboratory of Genetic and Metabolic Diseases in Children, Wuxi Children's Hospital, Affiliated Children's Hospital of Jiangnan University, Wuxi, Jiangsu, China; 4 Public Health Research Center, Department of Public Health and Preventive Medicine, Wuxi School of Medicine Jiangnan University, Wuxi, Jiangsu, China; 5 Department of Pediatric Surgery, Affiliated Children’s Hospital of Jiangnan University (Wuxi Children’s Hospital), Wuxi, Jiangsu, China

**Keywords:** obesity indices, MAFLD, children, Wuxi (China) Children’s Nutrition and Health cohort, screening performance

## Abstract

In recent years, as childhood obesity rates soar globally, there has been a concomitant increase in the prevalence of metabolic dysfunction-associated fatty liver disease (MAFLD). The intent of this analysis is to explore the correlation between four novel obesity indices and pediatric MAFLD, and to compare their varying screening performance for MAFLD. We used data from the Wuxi (China) Children’s Nutrition and Health cohort. Logistic regression and restricted cubic spline (RCS) were employed to assess the associations between the obesity indices, including visceral adiposity index (VAI), lipid accumulation product (LAP), body roundness index (BRI), and weight-adjusted waist index (WWI) with MAFLD in children. Furthermore, Receiver operating characteristic (ROC) curve analysis was used to evaluate the predictive performance of the four novel obesity indices, and DeLong’s test was applied to compare the AUC of the best-performing index (BRI) with those of traditional anthropometric measures (BMI, WC, and WHtR). A total of 1,214 children (aged 6–14 years) were enrolled in this study, with a mean age of 10.3 years and a boy proportion of 56.4%. Among them, 205 (16.8%) were diagnosed with MAFLD, while the remaining 1,009 (83.1%) served as controls. The multivariate logistic regression revealed that the VAI, LAP, BRI and WWI displayed a marked association with MAFLD in children (all *p* < 0.001). Among them, BRI exhibited high and consistent screening performance for pediatric MAFLD across all analyzed subgroups. For instance, in the subgroup with CAP ≥ 248 dB/m, it achieved an AUC of 0.929 (95% CI: 0.913–0.945), comparable to traditional BMI and WHtR, and significantly outperformed WC. Our findings demonstrated four the novel obesity indices are significantly associated with MAFLD in children. The BRI emerges as a simple, reliable, and clinically useful screening tool that captures visceral fat distribution and may aid in early risk stratification.

## 1 Introduction

Metabolic dysfunction-associated fatty liver disease (MAFLD) has emerged as the preeminent etiology underlying chronic liver disease in children and adolescents, primarily involving the accumulation of excess fat in hepatocytes (1). Formerly referred to as nonalcoholic fatty liver disease (NAFLD), the term MAFLD was proposed by an international panel of experts to better reflect the metabolic dysregulation underlying the disease (2–4). MAFLD is intimately associated with genetic predisposition, as well as epigenetic and various other contributing factors, such as obesity, lipodystrophic conditions, insulin resistance (IR), and metabolic syndrome (MetS) (5). It encompasses a wide range of liver pathologies, from benign hepatic steatosis to more severe steatohepatitis, which can progress to advanced fibrosis and ultimately cirrhosis (6). The MAFLD diagnostic criterion improves the ability to identify individuals at higher risk of developing cardiovascular, liver, and metabolic diseases, offering greater clinical utility compared to the former NAFLD definition (7). Data demonstrate that adolescents with MAFLD are at a higher risk of developing hypertension, metabolic syndrome, type 2 diabetes, chronic kidney disease, and cardiovascular diseases (8–10). When the production and/or intake of fatty acids in the liver outstrips its capacity for oxidation and/or excretion, lipid droplets accumulate within its tissue matrix, ultimately leading to MAFLD (11). As adolescent obesity rates soar globally, there has been a concomitant increase in the prevalence of MAFLD. In America, MAFLD prevalence ranged from 10 to 35%, with obese adolescents experiencing a notably higher prevalence, reaching up to 29 to 38% (12). However, identifying the high-risk subpopulation constitutes a pivotal challenge, as exhibit symptoms of MAFLD are often absent in most individuals.

Historically, the diagnosis of MAFLD has relied on liver biopsy (13), which is a painful and invasive procedure, posing significant challenges for early identification of MAFLD. Against this backdrop, a promising approach is the use of obesity measurement indices, which can reflect the degree of obesity and potentially serve as screening tools for MAFLD (14–17). These indices encompass both traditional measures, such as waist circumference (WC), body mass index (BMI), and waist-to-height ratio (WHtR), as well as novel indices introduced in recent years, which include visceral adiposity index (VAI), lipid accumulation product (LAP), body roundness index (BRI), and weight-adjusted waist index (WWI). While a few studies have explored the correlation between these novel obesity indices and NAFLD, shedding light on their screening value (18). However, in the field of MAFLD, particularly in pediatric, remains largely unexplored.

This study aims to examine the correlation between the four novel obesity indices and pediatric MAFLD, and to compare their differences in screening the risk of MAFLD.

## 2 Materials and methods

### 2.1 Study design and population

This cross-sectional study utilized data from the Wuxi Children’s Nutrition and Health cohort. The Wuxi (China) cohort study was conducted in Wuxi City, Jiangsu Province, China, with approximately 2,218 students aged 6–14 years recruited in two waves during March–April 2023 and March–April 2024 (19). All participants completed a routine elementary school physical examination to evaluate their nutritional and developmental status. These assessments included laboratory tests, vibration-controlled transient elastography (VCTE), and bioelectrical impedance analysis (20). Prior to the recruitment of participants, the study was prospectively registered at the Chinese Clinical Trials Registry (Approval No. ChiCTR2400080508) and approved by the Ethics Committee of the Affiliated Children’s Hospital of Jiangnan University (Approval no. WXCH2022-09-044). In addtion, written informed consent was obtained from the parents or legal guardians of all eligible children.

**Figure 1:**
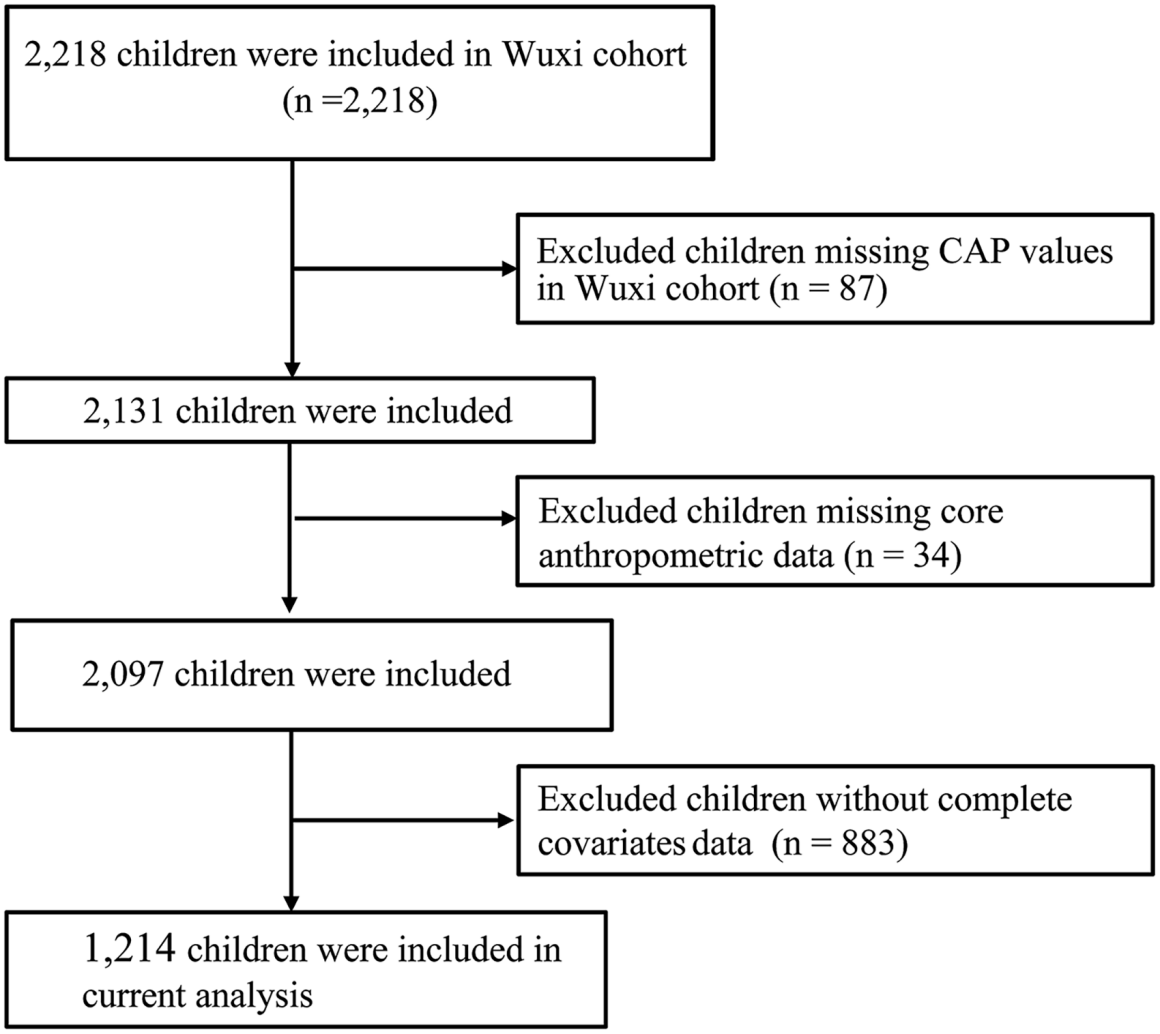
Flowchart of participants selection.

This study initially included all children aged 6–14 years from the Wuxi cohort, with a total of 2,218 children considered for analysis. Children were then excluded based on the following criteria: (1) children with missing data for the controlled attenuation parameter (CAP), which quantifies the degree of steatosis; (2) children missing core anthropometric data; (3) children with incomplete covariates data. Finally, a total of 1,214 children were enrolled in the study (Figure 1).

### 2.2 Definition of MAFLD

Following the international consensus definition for paediatric MAFLD (21), MAFLD was diagnosed in children with evidence of hepatic steatosis who also met at least one of the following three criteria: (1) overweight or obesity [according to the World Health Organization (WHO) age and sex-specific BMI reference standards] (22), or abdominal obesity defined by WC > the 90th percentile; (2) type 2 diabetes or prediabetes [defined as a prior diagnosis of diabetes, glycosylated hemoglobin (HbA1c) > 5.7%, or fasting blood glucose (GLU) ≥ 100 mg/dL]; or (3) evidence of metabolic dysregulation (21).

Metabolic dysregulation was further defined according to age-specific criteria based on the paediatric consensus (21). For children aged 6 to 9 years, it was identified by the presence of at least one of the following: elevated plasma triglyceride concentration > 90th percentile, low plasma HDL cholesterol concentration ≤ 10th percentile, or elevated systolic/diastolic blood pressure > 90th percentile. For children aged 10 to 15 years, the metabolic dysregulation was defined by the presence of at least two of the following abnormalities: (a) blood pressure > 130/85 mmHg; (b) triglycerides (TG) ≥ 150 mg/dL; (c) high-density lipoprotein (HDL)-cholesterol < 40 mg/dL; (d) a triglyceride-to-HDL-cholesterol ratio > 2.25 (21, 23).

Evidence of hepatic steatosis was quantified using the controlled attenuation parameter (CAP) measured by transient elastography (TE). In this study, elastography examinations were conducted using FibroScan devices by trained and experienced technicians. The exams were considered acceptable only if at least 10 valid measurements were obtained after a fasting time of at least 3 h, with an interquartile range/median < 30%. To enhance the diagnostic accuracy and reliability of our approach, we employed three predefined CAP thresholds (248 dB/m, 260 dB/m, and 280 dB/m) for steatosis quantification, thereby establishing an objective diagnosis of MAFLD (14, 23, 24).

Accordingly, children were assigned to the MAFLD group if they met both the steatosis criteria (CAP ≥ 248 dB/m) and at least one metabolic abnormality criterion. Children without evidence of hepatic steatosis (CAP < 248 dB/m) were assigned to the control group, regardless of whether they met any metabolic abnormality criteria (e.g., overweight/obesity, diabetes, or metabolic dysregulation).

### 2.3 Four novel obesity indices and laboratory measurement

In the Wuxi cohort, trained medical staff conducted school-based examinations following a standardized protocol. Measurements included height, weight, and blood pressure. Fasting (≥ 8 h) venous blood samples were collected for laboratory assays. We extracted demographic variables from the Wuxi cohort, including sex, age. In addition, we also extracted variables on height (cm), weight status, WC (cm), BMI (kg/m^2^), TG (mg/dL), HDL (mg/dL), total cholesterol (TCHOL, mg/dL), fasting GLU (mmol/L), fasting insulin (μU/mL), Alanine Aminotransferase (ALT, U/L), Aspartate Aminotransferase (AST, U/L) and blood pressure (mmHg). Weight status was categorized as normal weight, overweight, and obesity according to the World Health Organization (WHO) age- and sex-specific BMI reference standards [22]. Waist-to-height ratio (WHtR) was calculated as WC (cm) divided by height (cm). After extracting blood pressure three times, the final blood pressure values were derived by calculating the arithmetic mean of systolic blood pressure (SBP) and diastolic blood pressure (DBP) measurements.

The BRI was proposed by Thomas et al. (25) as an anthropometric index combining height and WC to estimate body fat distribution. Although originally developed in adults, this index has been subsequently validated in pediatric populations (26, 27). Park et al. (28) introduced the WWI as a novel anthropometric measure that adjusts WC for body weight, and its application in children has been explored in previous studies (29, 30). The VAI was proposed by Amato et al. (31) as a sex-specific indicator of visceral adipose tissue dysfunction, and its use has since been extended to pediatric populations (32, 33). Kahn et al. (34) described the Lipid Accumulation Product (LAP) as a simple index for estimating lipid overaccumulation in adults, which has also been investigated in children and adolescents in recent years (35, 36).

We calculated the indices based on the following mathematical expressions (25, 28, 31, 34):


$$\mathrm{BRI}=364.2-365.5\times {\left[1-{\left(\frac{WC_{\left(m\right)}}{2\pi }\right)}^{2}/{\left(\frac{\text{heigh}t_{\left(m\right)}}{2}\right)}^{2}\right]}^{\frac{1}{2}}$$

$$\mathrm{WWI}=WC_{\left(\mathrm{cm}\right)}/\sqrt{\text{weight}(\mathrm{kg})}$$

$$\mathrm{LA}P_{\left(\text{male}\right)}=WC_{\left(\mathrm{cm}\right)}-65\times TG_{\left(\text{mmol}/L\right)}$$

$$\mathrm{LA}P_{\left(\text{female}\right)}=WC_{\left(\mathrm{cm}\right)}-58\times TG_{\left(\text{mmol}/L\right)}$$

$$\begin{array}{l} \mathrm{VAI}(\text{male})=(\frac{WC_{\left(\mathrm{cm}\right)}}{39.68+(1.88\times \mathrm{BMI})})\times (\frac{TG_{\left(\text{mmol}/L\right)}}{1.03})\\ \times (\frac{1.31}{\mathrm{HD}L_{\left(\text{mmol}/L\right)}}) \end{array}$$

$$\begin{array}{l} \mathrm{VAI}(\text{female})=(\frac{WC_{\left(\mathrm{cm}\right)}}{36.58+(1.89\times \mathrm{BMI})})\times (\frac{TG_{\left(\text{mmol}/L\right)}}{0.81})\\ \times (\frac{1.52}{\mathrm{HD}L_{\left(\text{mmol}/L\right)}}) \end{array}$$

### 2.4 Statistical analysis

In the descriptive analysis, we utilized frequency (percentage) to summarize categorical variables, and for continuous variables exhibiting skewed distributions, we presented them using the median (25th, 75th). We then used *χ*^2^ test and wilcoxon rank-sum test to assess differences in sociodemographic and clinical characteristics between the MAFLD group and non-MAFLD group. We implemented multivariate logistic regression to assess the relationship between these obesity indices and MAFLD. Model A was a crude model; Model B was adjusted for age, and sex, as these were proposed as potential confounders. Then, restricted cubic spline (RCS) was employed to determine whether there was a nonlinear dose–response relationship of the obesity indices and MAFLD, with four knots placed at the 5th, 35th, 65th, and 95th percentile (37). Furthermore, the predictive performance of these indices for MAFLD in children was evaluated using the receiver operating characteristic (ROC) curve analysis and area under the curve (AUC), with calculations of optimal cutoff threshold (determined by Youden index), sensitivity, specificity, positive predictive value (PPV), and negative predictive value (NPV). Furthermore, pairwise comparisons of the area under the ROC curves (AUC) between BRI and traditional anthropometric indices (BMI, WC, and WHtR) were performed using the DeLong test to evaluate the statistical significance of differences in screening performance.

All analyses were performed using R (version 4.3.2). As a criterion for statistical significance, we adopted two-sided *p* < 0.05 as an indicator of statistical significance.

## 3 Results

### 3.1 Participant characteristics

This study included 1,214 children aged 6 to 14 years, with a mean age of 10.3 years and a boy proportion of 56.4%. Among them, 205 (16.9%) were diagnosed with MAFLD, while the remaining 1,009 (83.1%) served as controls. As shown in Table 1, compared to the control group, children with MAFLD had a significantly different distribution of weight status (*p* < 0.001), characterized by a markedly higher proportion of obesity (67.3% vs. 10.1%) and overweight (29.8% vs. 18.8%), and a lower proportion of normal weight (2.9% vs. 71.1%). WHtR was also significantly higher in the MAFLD group (0.51 vs. 0.43, *p* < 0.001). Furthermore, children with MAFLD were more likely to be male and had a significantly higher prevalence of diabetes (*p* < 0.001). In addition, they exhibited significantly higher levels of height, weight, WC, BMI, TG, fasting glucose, ALT, AST, SBP, DBP, and fasting insulin, while HDL-C levels were lower (all *p* < 0.05).

**Table 1:** Characteristics of study population (*N =* 1,214).

Characteristics	Overall	Non-MAFLD (*N =* 1,009)	MAFLD (*N =* 205)	*P*
*N* (%)	1,214 (100.0)	1,009 (83.1)	205 (16.9)	
Age (y)	10.3 (9.5, 11.5)	10.2 (9.4, 11.4)	11.3 (10.0, 12.4)	< 0.001
Sex				
Girl	529 (43.6)	479 (47.5)	50 (24.4)	< 0.001
Boy	685 (56.4)	530 (52.5)	155 (75.6)	
Height (cm)	145.0 (138.0, 153.5)	143.5 (137.5, 151.5)	153.0 (145.0, 161.0)	< 0.001
Weight status				
Normal	723 (59.5)	717 (71.1)	6 (2.9)	< 0.001
Overweight	251 (20.7)	190 (18.8)	61 (29.8)	
Obesity	240 (19.8)	102 (10.1)	138 (67.3)	
Waist (cm)	63.0 (58.5, 70.9)	61.4 (58.0, 66.8)	78.2 (72.4, 85.2)	< 0.001
WHtR	0.44 (0.42, 0.48)	0.43 (0.41, 0.45)	0.51 (0.48, 0.54)	< 0.001
BMI (kg/m^2^)	18.0 (16.1, 21.3)	17.3 (15.8, 19.7)	24.3 (22.2, 26.2)	< 0.001
TG (mg/dL)	65.5 (52.2, 84.1)	62.8 (50.5, 79.7)	84.1 (62.8, 111.5)	< 0.001
HDL (mg/dL)	58.0 (50.7, 65.7)	59.6 (51.8, 66.9)	50.67 (43.7, 58.4)	< 0.001
TCHOL (mg/dL)	173.6 (156.2, 193.0)	172.7 (156.2, 191.8)	179.8 (156.2, 198.4)	0.109
GLU (mmol/L)	4.7 (4.5, 4.9)	4.7 (4.5, 4.9)	4.89 (4.7, 5.1)	< 0.001
Insulin (μU/mL)	7.3 (5.1, 11.0)	6.6 (4.8, 9.6)	12.6 (8.4, 18.6)	< 0.001
ALT (U/L)	13.3 (11.1, 16.9)	12.8 (10.7, 15.4)	18.0 (14.0, 24.4)	< 0.001
AST (U/L)	25.0 (22.0, 28.0)	25.0 (22.0, 28.0)	24.0 (21.0, 27.0)	0.008
SBP (mmHg)	107.0 (99.0, 116.0)	106.0 (98.0, 114.0)	118.0 (109.8, 125.3)	< 0.001
DBP (mmHg)	64.0 (59.0, 70.0)	64.0 (59.0, 69.0)	69.0 (63.0, 74.0)	< 0.001
Diabetes/pre-diabetes				
No	1,202 (99.0)	1,005 (99.6)	197 (96.1)	< 0.001
Yes	12 (1.0)	4 (0.4)	8 (3.9)	

MAFLD, Metabolic dysfunction-associated fatty liver disease; WHtR, Waist-to-Height Ratio; BMI, body mass index; TG, triglyceride; HDL, high-density lipoprotein; TCHOL, total Cholesterol; GLU, fasting blood glucose; ALT, Alanine Aminotransferase; AST, Aspartate Aminotransferase; SBP, systolic blood pressure; DBP, diastolic blood pressure.

### 3.2 Associations between the four obesity indices and MAFLD in children

Table 2 presents the relationship between the four obesity indices and MAFLD risk by multivariate logistic regression. In the crude model (model A), significant positive associations were observed between all obesity indices and MAFLD risk (all *p* < 0.001). After adjustment for age and sex (Model B), the associations remained significant across CAP-defined subgroups. Specifically, in participants with CAP ≥ 248 dB/m, the adjusted odds ratios were as follows: VAI, 5.72 (95% CI: 3.85–8.70); LAP, 1.19 (95% CI: 1.13–1.26); BRI, 10.58 (95% CI: 8.06–14.18); and WWI, 4.87 (95% CI: 2.99–8.07). Similarly. these positive associations were consistently observed in the subgroups with CAP ≥ 260 dB/m and CAP ≥ 280 dB/m (all *p* < 0.001).

**Table 2:** The correlation between four novel obesity indices and MAFLD in children (*N =* 1,214).

Obesity indices	Model A		Model B	
	OR (95% CI)	*P*	OR (95% CI)	*P*
CAP ≥ 248 dB/m				
VAI	3.24 (2.48, 4.29)	< 0.001	5.72 (3.85, 8.70)	< 0.001
LAP	1.15 (1.12, 1.19)	< 0.001	1.19 (1.13, 1.26)	< 0.001
BRI	11.91 (9.08, 15.94)	< 0.001	10.58 (8.06, 14.18)	< 0.001
WWI	1.91 (1.34, 2.75)	< 0.001	4.87 (2.99, 8.07)	< 0.001
CAP ≥ 260 dB/m				
VAI	2.70 (2.06, 3.58)	< 0.001	3.94 (2.60, 6.16)	< 0.001
LAP	1.12 (1.10, 1.14)	< 0.001	1.14 (1.11, 1.16)	< 0.001
BRI	7.97 (6.33, 10.20)	< 0.001	6.79 (5.29, 8.86)	< 0.001
WWI	2.40 (1.57, 3.67)	< 0.001	6.98 (3.71, 13.62)	< 0.001
CAP ≥ 280 dB/m				
VAI	2.33 (1.74, 3.14)	< 0.001	2.72 (1.80, 4.15)	< 0.001
LAP	1.11 (1.09, 1.12)	< 0.001	1.11 (1.09, 1.13)	< 0.001
BRI	6.52 (5.15, 8.40)	< 0.001	5.22 (4.15, 6.68)	< 0.001
WWI	2.42 (1.39, 4.20)	< 0.001	7.18 (3.14, 17.42)	< 0.001

Model A, unadjusted model; Model B, adjusted for age, sex; MAFLD, metabolic dysfunction-associated fatty liver disease; VAI, visceral obesity index; LAP, lipid accumulation; BRI, body roundness index; WWI, weight-adjusted waist index. Bold values indicate statistical significance (*p* < 0.05).

### 3.3 Non-linear relationship between the four obesity indices and MAFLD in children

**Figure 2:**
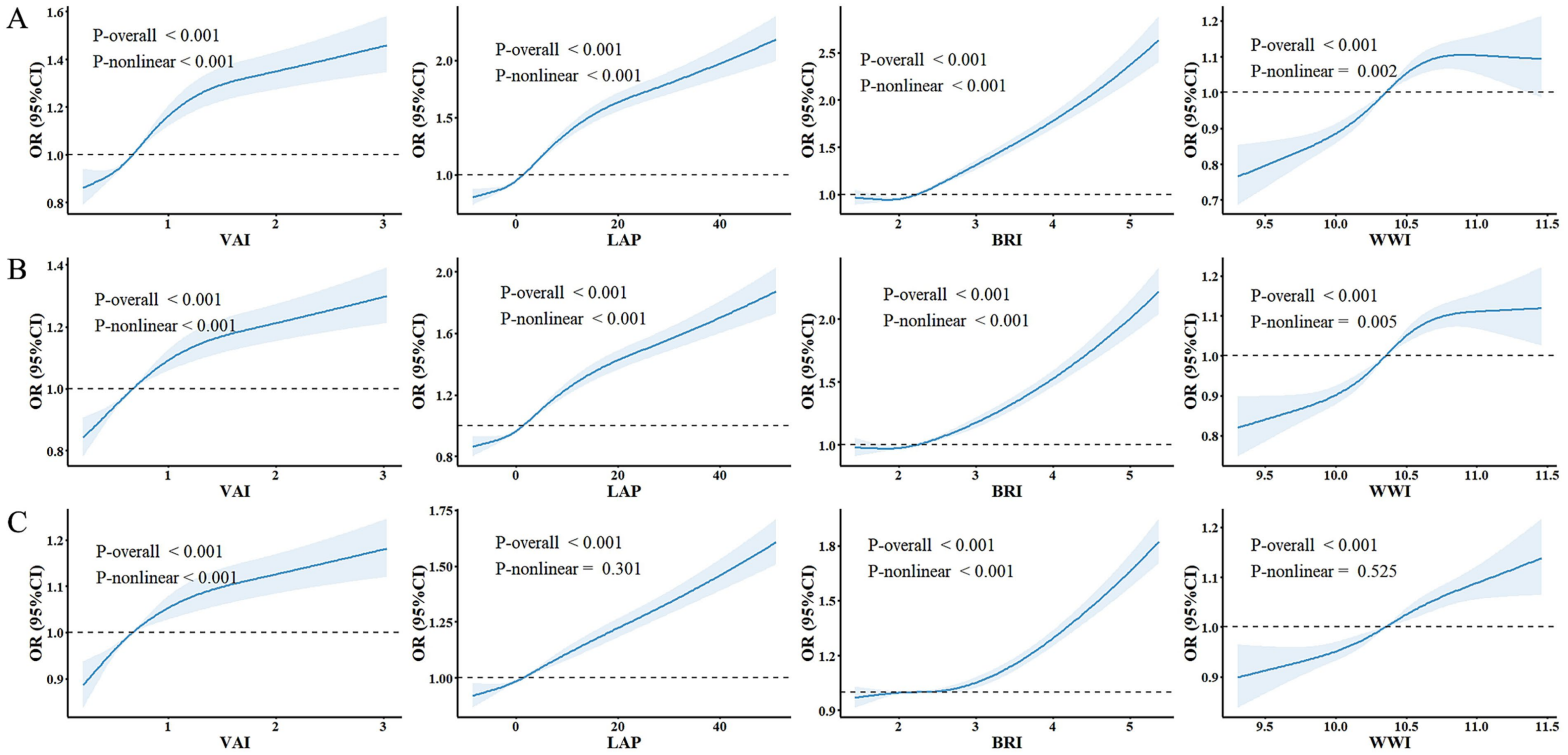
Restricted cubic spline (RCS) analysis for the association between four obesity indices and MAFLD in the Wuxi cohort population. The solid and dashed lines represent the OR and 95% CI. **(A)** MAFLD diagnosed using the CAP threshold of ≥ 248 dB/m. **(B)** MAFLD diagnosed using the CAP threshold of ≥ 260 dB/m. **(C)** MAFLD diagnosed using the CAP threshold of ≥ 280 dB/m.

RCS curves were employed to explore the relationships between the four novel obesity indices and MAFLD (Figure 2). After adjusting for age, and sex, significant nonlinear associations with MAFLD risk were observed for VAI, LAP, BRI, and WWI in the CAP ≥ 248 dB/m subgroup (*P*_nonlinear_ < 0.05). The nonlinear associations for VAI and BRI remained significant at the higher CAP thresholds (≥ 260 dB/m and ≥ 280 dB/m). However, LAP and WWI did not exhibit significant non-linearity particularly within the CAP ≥ 280 dB/m subgroup (*P*_nonlinear_ > 0.05).

### 3.4 ROC analysis for the obesity indices and their combined indices for MAFLD in children

**Figure 3:**
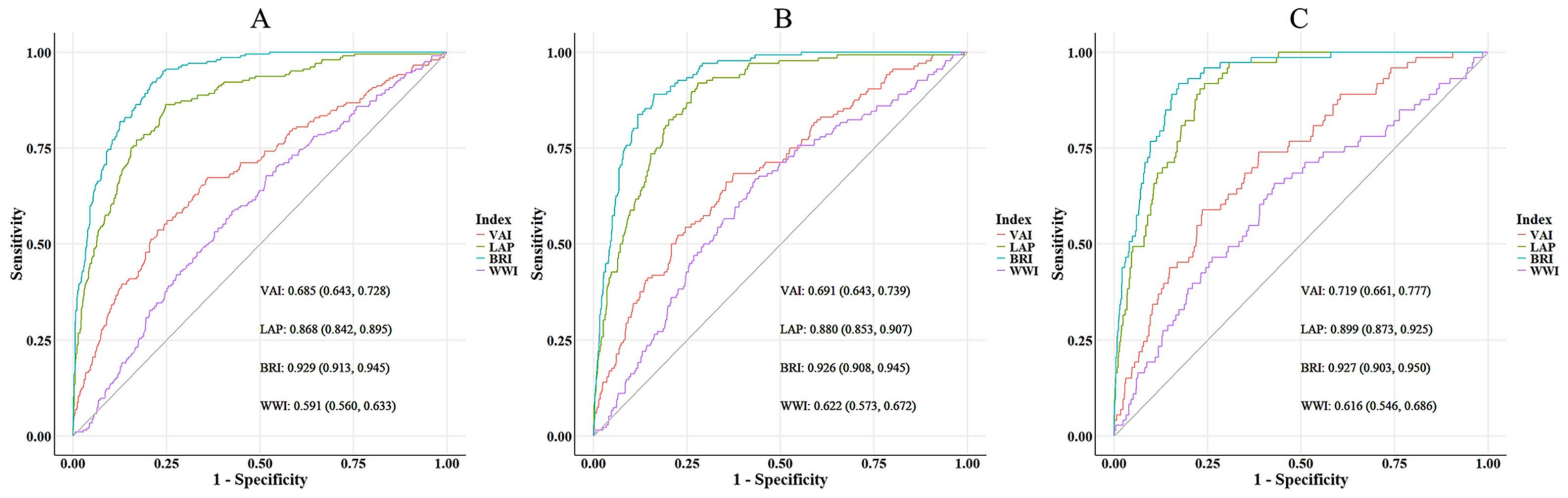
Receiver operating characteristic (ROC) analysis for the four obesity indices in the Wuxi cohort population and stratified by CAP thresholds. **(A)** MAFLD diagnosed using the CAP threshold of ≥ 248 dB/m. **(B)** MAFLD diagnosed using the CAP threshold of ≥ 260 dB/m. **(C)** MAFLD diagnosed using the CAP threshold of ≥ 280 dB/m.

We further assessed the screening performance of the four obesity indices for MAFLD using ROC analysis (Table 3 and Figure 3). The results showed that BRI demonstrated significantly better screening capability for MAFLD than other indices in the subgroup with CAP ≥ 248 dB/m (AUC: 0.929; 95%CI: 0.913–0.945). The optimal cutoff threshold selected was a predictive value of 0.079, with a sensitivity of 95.1%, specificity of 76.0%, PPV of 44.6%, NPV of 98.7%, and Youden index of 0.711. These associations were largely consistent across the subgroups with CAP ≥ 260 dB/m and CAP ≥ 280 dB/m.

**Table 3:** The diagnostic performance of four novel obesity indices for MAFLD in children (*N =* 1,214).

Obesity indices	AUC (95%CI)	Best threshold	PPV	NPV	Sensitivity	Specificity	Youden index
CAP ≥ 248 dB/m							
VAI	0.685 (0.643, 0.728)	0.149	0.276	0.906	0.673	0.641	0.314
LAP	0.868 (0.842, 0.895)	0.117	0.415	0.964	0.863	0.752	0.615
BRI	0.929 (0.913, 0.945)	0.079	0.446	0.987	0.951	0.760	0.711
WWI	0.591 (0.560, 0.633)	0.162	0.211	0.881	0.678	0.484	0.162
CAP ≥ 260 dB/m							
VAI	0.691 (0.643, 0.739)	0.098	0.188	0.940	0.684	0.626	0.310
LAP	0.880 (0.853, 0.907)	0.072	0.293	0.986	0.919	0.721	0.640
BRI	0.926 (0.908, 0.945)	0.103	0.410	0.984	0.890	0.839	0.729
WWI	0.622 (0.573, 0.672)	0.100	0.163	0.932	0.669	0.568	0.237
CAP ≥ 280 dB/m							
VAI	0.719 (0.661, 0.777)	0.061	0.137	0.967	0.589	0.763	0.352
LAP	0.899 (0.873, 0.925)	0.042	0.195	0.993	0.918	0.758	0.676
BRI	0.927 (0.903, 0.950)	0.052	0.254	0.994	0.918	0.827	0.745
WWI	0.616 (0.546, 0.686)	0.059	0.089	0.963	0.657	0.571	0.228

MAFLD, metabolic dysfunction-associated fatty liver disease; VAI, visceral obesity index; LAP, lipid accumulation; BRI, body roundness index; WWI, weight-adjusted waist index; PPV, positive predictive value; NPV, negative predictive value.

To explore whether combined indices could enhance MAFLD predictive performance, we combined VAI, LAP, BRI, and WWI in pairs and constructed six combination indices, including VAI_LAP, VAI_BRI, VAI_WWI, LAP_BRI, LAP_WWI, and BRI_WWI. The predictive performance of these combined indices for MAFLD was evaluated using ROC curves, with results stratified by the same CAP subgroups (CAP ≥ 248 dB/m, ≥ 260 dB/m, and ≥ 280 dB/m) as the single index analysis. However, as shown in Table 4, across all analyzed CAP subgroups, none of the paired combination indices exhibited statistically superior predictive performance compared to the best-performing single index, BRI.

**Table 4:** The diagnostic performance of six combined indices for MAFLD in children (*N =* 1,214).

Combined indices	AUC (95%CI)	Best threshold	PPV	NPV	Sensitivity	Specificity	Youden index
CAP ≥ 248 dB/m							
VAI_LAP	0.877 (0.850, 0.903)	0.153	0.487	0.953	0.800	0.829	0.629
VAI_BRI	0.931 (0.916, 0.946)	0.095	0.468	0.983	0.932	0.785	0.717
VAI_WWI	0.693 (0.652, 0.735)	0.153	0.269	0.902	0.659	0.636	0.295
LAP_BRI	0.931 (0.915–0.946)	0.098	0.471	0.980	0.922	0.790	0.712
LAP_WWI	0.869 (0.842, 0.896)	0.120	0.429	0.963	0.854	0.769	0.623
BRI-WWI	0.932 (0.917, 0.948)	0.138	0.515	0.975	0.898	0.829	0.727
CAP ≥ 260 dB/m							
VAI_LAP	0.890 (0.861, 0.918)	0.094	0.375	0.980	0.868	0.817	0.685
VAI_BRI	0.928 (0.910, 0.946)	0.130	0.462	0.978	0.846	0.876	0.722
VAI_WWI	0.712 (0.667, 0.758)	0.092	0.176	0.947	0.757	0.554	0.311
LAP_BRI	0.928 (0.910, 0.946)	0.135	0.469	0.978	0.846	0.879	0.725
LAP_WWI	0.883 (0.856, 0.911)	0.075	0.318	0.987	0.919	0.751	0.670
BRI-WWI	0.928 (0.910, 0.946)	0.151	0.496	0.982	0.868	0.889	0.757
CAP ≥ 280 dB/m							
VAI_LAP	0.916 (0.890, 0.942)	0.044	0.227	0.993	0.918	0.800	0.718
VAI_BRI	0.928 (0.905, 0.950)	0.053	0.265	0.993	0.904	0.840	0.744
VAI_WWI	0.713 (0.649, 0.776)	0.071	0.154	0.965	0.548	0.808	0.356
LAP_BRI	0.929 (0.907, 0.951)	0.052	0.274	0.993	0.904	0.847	0.751
LAP_WWI	0.899 (0.871, 0.926)	0.036	0.181	0.996	0.959	0.723	0.682
BRI-WWI	0.939 (0.922, 0.956)	0.061	0.289	0.996	0.945	0.851	0.796

MAFLD, metabolic dysfunction-associated fatty liver disease; PPV, positive predictive value; NPV, negative predictive value.

### 3.5 Comparison of BRI with traditional anthropometric measures

To directly benchmark BRI against traditional anthropometric measures, we compared its predictive performance with that of BMI, WC, and WHtR using ROC analysis across three CAP thresholds (≥ 248, ≥ 260, and ≥ 280 dB/m). The results are presented in Table 5. BRI demonstrated high and stable AUCs (0.926–0.929) across all thresholds, comparable to those of BMI (0.925–0.937) and WHtR (0.926–0.928). DeLong’s test confirmed no significant differences between BRI and BMI or between BRI and WHtR at any threshold (DeLong test, all *p* > 0.05). Furthermore, in the CAP ≥ 248 dB/m subgroup, BRI achieved a significantly higher AUC than WC (0.929 vs. 0.908, DeLong test, *p* < 0.001), whereas no significant differences were observed at higher CAP thresholds (DeLong test, both *p* > 0.05).

**Table 5:** Comparative evaluation of BRI and traditional obesity indices for MAFLD in children (*N =* 1,214).

Obesity indices	AUC (95% CI)	Best threshold	PPV	NPV	Sensitivity	Specificity	Youden index
CAP ≥ 248 dB/m							
BRI	0.929 (0.913, 0.945)	0.079	0.446	0.987	0.951	0.760	0.711
BMI	0.929 (0.914, 0.945)	0.113	0.482	0.983	0.932	0.797	0.729
WC	0.908 (0.887, 0.929)	0.121	0.470	0.968	0.868	0.801	0.669
WHtR	0.928 (0.913, 0.944)	0.092	0.449	0.982	0.932	0.768	0.700
CAP ≥ 260 dB/m							
BRI	0.926 (0.908, 0.945)	0.103	0.410	0.984	0.890	0.839	0.729
BMI	0.925 (0.906, 0.944)	0.147	0.468	0.981	0.868	0.876	0.744
WC	0.916 (0.892, 0.939)	0.131	0.451	0.979	0.853	0.869	0.722
WHtR	0.926 (0.908, 0.944)	0.146	0.469	0.977	0.838	0.880	0.718
CAP ≥ 280 dB/m							
BRI	0.927 (0.903, 0.950)	0.052	0.254	0.994	0.918	0.827	0.745
BMI	0.937 (0.920, 0.955)	0.063	0.291	0.995	0.945	0.853	0.798
WC	0.934 (0.9158, 0.953)	0.052	0.261	0.994	0.917	0.833	0.750
WHtR	0.927 (0.904, 0.949)	0.060	0.267	0.992	0.890	0.844	0.734

Pairwise comparisons of AUCs were performed using DeLong’s test. No significant differences were observed between BRI and BMI or between BRI and WHtR at any CAP threshold (all *P* > 0.05). For BRI versus WC, a statistically significant difference was observed only at the CAP ≥248 dB/m threshold (*P* < 0.001); at higher thresholds (≥260 and ≥280 dB/m), the differences were not significant (*P* > 0.05). MAFLD, metabolic dysfunction-associated fatty liver disease; BRI, body roundness index; PPV, positive predictive value; NPV, negative predictive value.

## 4 Discussion

This study evaluated the associations between several novel obesity indices and MAFLD in a pediatric cohort from Wuxi, China. Our findings demonstrated that VAI, LAP, BRI, and WWI were each significantly associated with an increased risk of pediatric MAFLD. ROC analyses demonstrates that among four novel obesity indices, BRI shows the highest screening performance for pediatric MAFLD, with accuracy comparable to traditional BMI and WHtR. Moreover, BRI outperforms WC in identifying mild hepatic steatosis. These findings support BRI as a simple, reliable, and clinically useful screening tool that captures visceral fat distribution and may aid in early risk stratification.

Research has indicated a tight correlation between the emergence and progression of MAFLD and disturbances in lipid metabolism, specifically those stemming from the accumulation of visceral adipose tissue, which contribute to dyslipidemia (38). Additionally, multiple studies have identified robust associations between MAFLD and various anthropometric measures, such as BMI, WC and WHtR (39, 40). Therefore, we selected four novel obesity indices to investigate their associations with MAFLD, including BRI, LAP, WWI, and VAI, which are calculated from simple anthropometric variables and lipid metabolism-related variables such as sex, TG, HDL-C, WC, BMI, weight. This is to our knowledge the few studies to access the relationship between multiple novel obesity indices and adolescent’s MAFLD, and to compare the screening performance of these indices. In the study, we found that the four novel obesity indices exhibited moderate to high screening performance for identifying children with MAFLD. The ROC analysis revealed that among the four novel indices, BRI demonstrated the highest screening capability for MAFLD in children. Notably, BRI demonstrated the highest AUC among the novel indices, and its performance was comparable to that of BMI and WHtR, with no statistically significant differences (DeLong test, all *p* > 0.05). Moreover, at the lowest CAP threshold (≥248 dB/m), BRI significantly outperformed WC (AUC: 0.929 vs. 0.908, *p* < 0.05), suggesting enhanced sensitivity for detecting milder hepatic steatosis. However, there are a few inconsistent with previous studies. Li et al. (41) conducted a study involving 4,195 adults and found that LAP has the potential to be a sensitive marker for detecting MAFLD among American adults. Liu et al. (42) carried out a study including 1,058 Chinese participants and identified fatty liver index (FLI) as the optimal screening marker for MAFLD. However, it is noteworthy to mention that the participants in these two studies were exclusively adults (average age approximately 40 years), and there is a lack of research involving child-specific cohorts.

MAFLD is primarily a chronic liver disease that arises as a consequence of hepatic lipid accumulation. Hepatic fat accumulation results from an imbalance in lipid acquisition and disposal, mediated by four key mechanisms: lipid uptake, *de novo* lipogenesis (DNL), fatty acid oxidation (FAO), and VLDL exportation (38). We demonstrated that BRI is a reliable screening tool for pediatric MAFLD. Its superior performance was validated across all subgroups, yielding AUCs of 0.929 (95% CI: 0.913–0.945) in the subgroup with CAP ≥ 248 dB/m. This finding underscores its utility as a reliable screening tool for children populations. One possible explanation is that BRI serves as a tool for measuring body fat and visceral fat. It not only reflects body roundness in relation to height, but also provides more accurate estimates of body fat percentage and visceral fat percentage. BRI also takes waist circumference into account, thus enabling a more comprehensive reflection of visceral fat distribution. However, the massive breakdown of visceral fat will increase gluconeogenesis in the liver, thereby reducing the output of VLDL, which can lead to impaired glucose tolerance and elevated TG levels (25, 43–45). Another plausible explanation comes from Mirzababaei et al.’s observational study (46), which demonstrated significant associations between BRI and cardiometabolic parameters (TG, HDL-C, LDL-C, BP, and FBS) along with inflammatory markers. During inflammatory states, adipose tissue initiates the release of fat and manufactures cortisol alongside pro-inflammatory cytokines, notably adipokines, with these factors collectively impacting metabolic health (44, 47).

The direct comparison between BRI and traditional anthropometric measures further supports its clinical utility. Although BMI is the most widely applied obesity index, it cannot distinguish between fat and lean mass, nor does it reflect fat distribution. In contrast, BRI is closely linked to visceral adiposity, a key driver in the pathogenesis of MAFLD. Our finding that BRI shows comparable performance to BMI and WHtR, and superior performance to WC, suggests that BRI serves as a simple, non-invasive alternative for pediatric MAFLD screening, particularly in settings requiring rapid assessment of central obesity.

VAI and LAP are important non-invasive biomarkers for visceral obesity and MAFLD, include triglycerides in their calculations. Previous studies conducted by Zou et al. and Guo et al. reported AUCs of 0.834 (95% CI: 0.825–0.843) and 0.854 (95% CI: 0.843–0.864), respectively, for LAP in detecting MAFLD. We found that LAP achieved an AUC of 0.868 (95% CI: 0.842–0.895) in the subgroup with CAP ≥ 248 dB/m, further validating LAP’s screening utility. Similarly, the AUC values of VAI in the two previous research reports are generally consistent with the results of our current study (48, 49). Collectively, these results highlight the potential of both LAP and VAI for the assessment of MAFLD. The potential explanation could be attributed to the harmful effects of dyslipidemia on hepatic sinusoidal endothelial cells, which leads to the translocation of chylomicron remnants to hepatocytes for the synthesis of very low-density lipoprotein (38). Furthermore, dyslipidemia can also compromise the capability of hepatic sinusoidal endothelial cells to synthesize nitric oxide, a recognized hepatic protective factor, due to its regulatory role in fat production and enhancement of fatty acid *β*-oxidation (50). It is remarkable that our research findings suggest that LAP possesses enhanced screening ability in comparison to VAI, and LAP’s simple computation utilizing WC and TG makes it a feasible and universally applicable instrument in clinical practice. Park et al. proposed a new anthropometric index, WWI, which assesses obesity by utilizing WC adjusted for body weight (28). Our study found a marked association between WWI and MAFLD, which was generally consistent with the previous finding by Shen et al. (17).

Additionally, in our study, we used TE to diagnose MAFLD in children. It is a widely used non-invasive method for assessing liver fibrosis and steatosis. However, several studies have raised concerns about the repeatability and precision of TE in pediatric populations. As highlighted by Kwon et al. (51), TE demonstrates significant inter-measurement variability in pediatric populations, particularly due to children smaller body habitus and narrower intercostal spaces. Another study by Ferraioli et al. found that while TE has some advantages in assessing liver steatosis in children, its precision and diagnostic accuracy need further validation (52). Furthermore, the CAP thresholds used for diagnosing MAFLD are primarily based on adult studies, and the diagnostic performance of these thresholds in children is yet to be fully established. Given these limitations, our results should be interpreted with caution. The diagnostic efficacy of TE in our study, while promising, may not fully reflect the true prevalence and severity of MAFLD in the pediatric population. The international expert consensus states that ultrasonography and ALT have similar, but moderate, diagnostic accuracy, and that combining both modalities improves sensitivity (21). Therefore, to address these diagnostic challenges, future research should prioritize the combined use of ultrasound and ALT in accordance with these recommendations, alongside large-scale multicenter studies aimed at establishing age-specific CAP thresholds for pediatric MAFLD.

The primary advantage of this study is that, to our knowledge, it is the first to comprehensively evaluate and compare the performance of four novel obesity indices (BRI, WWI, VAI, and LAP) in predicting pediatric MAFLD. Through systematic comparison of these indices, we demonstrated strong correlations between each index and MAFLD in children. Moreover, among the four novel indices, BRI exhibited the highest screening performance for pediatric MAFLD, with performance comparable to traditional BMI and WHtR and significantly better than WC in identifying milder hepatic steatosis. However, several limitations warrant attention. Firstly, while CAP values from VCTE may have limitations in diagnosing hepatic steatosis compared to liver biopsy (the gold standard), the invasive nature of biopsy makes it impractical for pediatric population. Nevertheless, our analyses using multiple CAP thresholds (≥ 248 dB/m, ≥ 260 dB/m, and ≥ 280 dB/m) demonstrated consistent and reliable results, supporting the robustness of our findings. Secondly, the school-based recruitment was conducted exclusively in Wuxi, a single urban city in China, which may limit the generalizability of our findings to other regions or populations with different ethnic, socioeconomic, or environmental backgrounds. Multi-center studies involving diverse geographic areas are warranted to validate our results. Third, although children with metabolic risk factors (e.g., diabetes/prediabetes) but without hepatic steatosis were included in the control group per the consensus definition, we acknowledge that they represent a high-risk population for future MAFLD, warranting longitudinal follow-up. Finally, although we controlled for some covariates that were considered relevant confounders, it is possible that there was still residual and unmeasured confounding (such as pubertal stage, diet, physical activity, SES, family history, inflammatory markers), which could introduce bias. In addition, the exclusion of a substantial number of participants due to missing data may introduce potential bias. Future studies should incorporate comprehensive assessments of these confounders and optimize data collection strategies to minimize bias.

## 5 Conclusion

This study demonstrates that four novel obesity indices exhibit significant associations with MAFLD in children. Among the four novel indices, BRI exhibited the highest screening performance for pediatric MAFLD, with performance comparable to traditional BMI and WHtR and significantly better than WC in identifying milder hepatic steatosis. These findings support BRI as a simple, reliable, and clinically useful screening tool that captures visceral fat distribution and may aid in early risk stratification.

## Data Availability

The raw data supporting the conclusions of this article will be made available by the authors, without undue reservation.

## References

[1] Chacón C Arteaga I Martínez-Escudé A Ruiz Rojano I Lamonja-Vicente N Caballeria L . Clinical epidemiology of non-alcoholic fatty liver disease in children and adolescents. The LiverKids: study protocol. PLoS One. (2023) 18:e0286586. doi: 10.1371/journal.pone.0286586, 37831682 PMC10575486

[2] Rinella ME Lazarus JV Ratziu V Francque SM Sanyal AJ Kanwal F . A multisociety Delphi consensus statement on new fatty liver disease nomenclature. J Hepatol. (2023) 79:1542–56. doi: 10.1016/j.jhep.2023.06.003, 37364790

[3] Brennan PN Tavabie OD Li W Marjot T Corless L Fallowfield JA . Progress is impossible without change: understanding the evolving nomenclature of steatotic liver disease and its effect on hepatology practice. Lancet Gastroenterol Hepatol. (2024) 9:577–82. doi: 10.1016/S2468-1253(23)00453-3, 38428439

[4] Eslam M Newsome PN Sarin SK Anstee QM Targher G Romero-Gomez M . A new definition for metabolic dysfunction-associated fatty liver disease: an international expert consensus statement. J Hepatol. (2020) 73:202–9. doi: 10.1016/j.jhep.2020.03.039, 32278004

[5] Kokkorakis M Boutari C Katsiki N Mantzoros CS. From non-alcoholic fatty liver disease (NAFLD) to steatotic liver disease (SLD): an ongoing journey towards refining the terminology for this prevalent metabolic condition and unmet clinical need. Metab Clin Exp. (2023) 147:155664. doi: 10.1016/j.metabol.2023.155664, 37517792

[6] Sweeny KF Lee CK. Nonalcoholic fatty liver disease in children. Gastroenterol Hepatol. (2021) 17:579–87. 35465068 PMC9021174

[7] Cheng YM Wang CC Kao JH. Metabolic associated fatty liver disease better identifying patients at risk of liver and cardiovascular complications. Hepatol Int. (2023) 17:350–6. doi: 10.1007/s12072-022-10449-x, 36471232

[8] Vittorio J Lavine JE. Recent advances in understanding and managing pediatric nonalcoholic fatty liver disease. F1000Res. (2020) 9:24198. doi: 10.12688/f1000research.24198.1, 32509277 PMC7238455

[9] Harlow KE Africa JA Wells A Belt PH Behling CA Jain AK . Clinically actionable hypercholesterolemia and hypertriglyceridemia in children with nonalcoholic fatty liver disease. J Pediatr. (2018) 198:76–83.e2. doi: 10.1016/j.jpeds.2018.02.038, 29661561 PMC6019181

[10] Zhang L El-Shabrawi M Baur LA Byrne CD Targher G Kehar M . An international multidisciplinary consensus on pediatric metabolic dysfunction-associated fatty liver disease. Med. (2024) 5:797–815.e2. doi: 10.1016/j.medj.2024.03.017, 38677287

[11] Souza-Mello V. Peroxisome proliferator-activated receptors as targets to treat non-alcoholic fatty liver disease. World J Hepatol. (2015) 7:1012–9. doi: 10.4254/wjh.v7.i8.1012, 26052390 PMC4450178

[12] Ciardullo S Monti T Perseghin GJC. Hepatology prevalence of liver steatosis and fibrosis detected by transient elastography in adolescents in the 2017–2018 National Health and nutrition examination survey (2021) 19:384–90. doi: 10.1016/j.cgh.2020.06.048 32623006

[13] Berger D Desai V Janardhan S. Con: liver biopsy remains the gold standard to evaluate fibrosis in patients with nonalcoholic fatty liver disease. Clinical Liver Dis. (2019) 13:114–6. doi: 10.1002/cld.740, 31061705 PMC6491029

[14] Zeng J Jin Q Yang J Yang RX Zhang RN Zhao J . Prevalence and incidence of MAFLD and associated anthropometric parameters among prepubertal children of the Shanghai birth cohort. Hepatol Int. (2023) 17:1416–28. doi: 10.1007/s12072-023-10574-1, 37728728

[15] Colantoni A Bucci T Cocomello N Angelico F Ettorre E Pastori D . Lipid-based insulin-resistance markers predict cardiovascular events in metabolic dysfunction associated steatotic liver disease. Cardiovasc Diabetol. (2024) 23:175. doi: 10.1186/s12933-024-02263-6, 38769519 PMC11106932

[16] Zhao E Wen X Qiu W Zhang C. Association between body roundness index and risk of ultrasound-defined non-alcoholic fatty liver disease. Heliyon. (2024) 10:e23429. doi: 10.1016/j.heliyon.2023.e23429, 38170062 PMC10758814

[17] Shen Y Wu Y Fu M Zhu K Wang J. Association between weight-adjusted-waist index with hepatic steatosis and liver fibrosis: a nationally representative cross-sectional study from NHANES 2017 to 2020. Front Endocrinol. (2023) 14:1159055. doi: 10.3389/fendo.2023.1159055, 37274346 PMC10235694

[18] Yetim A Şahin M Kandemir İ Bulakçı B Aksakal MT Karapınar E . Evaluation of the ability of insulin resistance and lipid-related indices to predict the presence of NAFLD in obese adolescents. Lipids Health Dis. (2024) 23:208. doi: 10.1186/s12944-024-02144-7, 38956572 PMC11218074

[19] Yang F Hu M Zhang H Zheng X Chen L Zhu L . Protocol for a longitudinal cohort study to understand characteristics and risk factors underlying vibration-controlled transient Elastography-diagnosed metabolic dysfunction-associated fatty liver disease children. Diabetes Metab. Syndrome Obesity Targets Ther. (2024) 17:4627–39. doi: 10.2147/DMSO.S492809, 39649758 PMC11625434

[20] Hu MY Sun DQ Yang F Zheng XW Wu NX Zhang HY . Impact of segmental body composition on metabolic dysfunction-associated fatty liver disease in Chinese children. Front Endocrinol. (2025) 16:1505050. doi: 10.3389/fendo.2025.1505050, 40034234 PMC11872705

[21] Eslam M Alkhouri N Vajro P Baumann U Weiss R Socha P . Defining paediatric metabolic (dysfunction)-associated fatty liver disease: an international expert consensus statement. Lancet Gastroenterol Hepatol. (2021) 6:864–73. doi: 10.1016/S2468-1253(21)00183-7, 34364544

[22] Child Growth Standards (2025). Available online at: https://www.who.int/toolkits/child-growth-standards/standards/body-mass-index-for-age-bmi-for-age

[23] Ciardullo S Monti T Perseghin G. Prevalence of liver steatosis and fibrosis detected by transient Elastography in adolescents in the 2017-2018 National Health and nutrition examination survey. Clin. Gastroenterol. Hepatol. Off. Clin. Prac. J. Am. Gastroenterol. Assoc. (2021) 19:384–390.e1. doi: 10.1016/j.cgh.2020.06.048, 32623006

[24] Karlas T Petroff D Sasso M Fan JG Mi YQ de Lédinghen V . Individual patient data meta-analysis of controlled attenuation parameter (CAP) technology for assessing steatosis. J Hepatol. (2017) 66:1022–30. doi: 10.1016/j.jhep.2016.12.022, 28039099

[25] Thomas DM Bredlau C Bosy-Westphal A Mueller M Shen W Gallagher D . Relationships between body roundness with body fat and visceral adipose tissue emerging from a new geometrical model. Obesity. (2013) 21:2264–71. doi: 10.1002/oby.20408, 23519954 PMC3692604

[26] Jahan A Abdullah MM Frank R Castellanos LJ Singer P Shen CL . Body roundness index is a stronger predictor of Cardiometabolic risk than body mass index in children between ages 8 to 17 years. J Pediatr. (2026) 288:114826. doi: 10.1016/j.jpeds.2025.114826, 40972710

[27] Dutta A Dutta PK Baruah SM Dihingia P Ray A Bhat DS . Non-autoimmune diabetes in young people from Assam, India: the PHENOEINDY-2 study. Diabetologia. (2025) 68:2179–93. doi: 10.1007/s00125-025-06500-9, 40751096

[28] Park Y Kim NH Kwon TY Kim SG. A novel adiposity index as an integrated predictor of cardiometabolic disease morbidity and mortality. Sci Rep. (2018) 8:16753. doi: 10.1038/s41598-018-35073-4, 30425288 PMC6233180

[29] Cui X Huang Y Kang L Han L Sun W Han K . A positive relationship between weight-adjusted waist index and non-alcoholic fatty liver disease: a study on US adolescents. Front Med. (2024) 11:1424667. doi: 10.3389/fmed.2024.1424667, 39845834 PMC11753237

[30] Sun P Zhang F Bi C Yin X Guo Y Hong J . Sex comparison of the association between weight-adjusted waist index and physical fitness index: a cross-sectional survey of adolescents in Xinjiang, China. Sci Rep. (2025) 15:18723. doi: 10.1038/s41598-025-03131-3, 40437112 PMC12119831

[31] Amato MC Giordano C Galia M Criscimanna A Vitabile S Midiri M . Visceral adiposity index: a reliable indicator of visceral fat function associated with cardiometabolic risk. Diabetes Care. (2010) 33:920–2. doi: 10.2337/dc09-1825, 20067971 PMC2845052

[32] Vizzuso S Del Torto A Dilillo D Calcaterra V Di Profio E Leone A . Visceral adiposity index (VAI) in children and adolescents with obesity: no association with daily energy intake but promising tool to identify metabolic syndrome (MetS). Nutrients. (2021) 13:413. doi: 10.3390/nu13020413, 33525454 PMC7911630

[33] Jakubiak GK Badicu G Surma S Waluga-Kozłowska E Chwalba A Pawlas N. The visceral adiposity index and its usefulness in the prediction of Cardiometabolic disorders. Nutrients. (2025) 17:2374. doi: 10.3390/nu17142374, 40732999 PMC12298961

[34] Kahn HS. The "lipid accumulation product" performs better than the body mass index for recognizing cardiovascular risk: a population-based comparison. BMC Cardiovasc Disord. (2005) 5:26. doi: 10.1186/1471-2261-5-26, 16150143 PMC1236917

[35] Zhang L Zhang Z Wang B Yuan Y Sun L Gao H . Relative children's lipid accumulation product is a novel Indicator for metabolic syndrome. Front Endocrinol. (2021) 12:645825. doi: 10.3389/fendo.2021.645825, 34093432 PMC8173219

[36] Chen ZY Liu L Zhuang XX Zhang YC Ma YN Liu Y . Lipid accumulation product is a better predictor of metabolic syndrome in Chinese adolescents: a cross-sectional study. Front Endocrinol. (2023) 14:1179990. doi: 10.3389/fendo.2023.1179990, 37424867 PMC10326626

[37] Zhao Y Gu Y Zhang B. Associations of triglyceride-glucose (TyG) index with chest pain incidence and mortality among the U.S. population. Cardiovasc Diabetol. (2024) 23:111. doi: 10.1186/s12933-024-02209-y, 38555461 PMC10981836

[38] Ipsen DH Lykkesfeldt J Tveden-Nyborg P. Molecular mechanisms of hepatic lipid accumulation in non-alcoholic fatty liver disease. CMLS. (2018) 75:3313–27. doi: 10.1007/s00018-018-2860-6, 29936596 PMC6105174

[39] Cai J Lin C Lai S Liu Y Liang M Qin Y . Waist-to-height ratio, an optimal anthropometric indicator for metabolic dysfunction associated fatty liver disease in the Western Chinese male population. Lipids Health Dis. (2021) 20:145. doi: 10.1186/s12944-021-01568-9, 34706716 PMC8549212

[40] Taheri E Moslem A Mousavi-Jarrahi A Hatami B Pourhoseingholi MA Asadzadeh Aghdaei H . Predictors of metabolic-associated fatty liver disease (MAFLD) in adults: a population-based study in northeastern Iran. Gastroenterol Hepatol Bed Bench. (2021) 14:S102–s111. 35154609 PMC8817755

[41] Li H Zhang Y Luo H Lin R. The lipid accumulation product is a powerful tool to diagnose metabolic dysfunction-associated fatty liver disease in the United States adults. Front Endocrinol. (2022) 13:977625. doi: 10.3389/fendo.2022.977625, 36407325 PMC9672518

[42] Liu J Duan S Wang C Wang Y Peng H Niu Z . Optimum non-invasive predictive indicators for metabolic dysfunction-associated fatty liver disease and its subgroups in the Chinese population: A retrospective case-control study. Front Endocrinol. (2022) 13:1035418. doi: 10.3389/fendo.2022.1035418, 36531447 PMC9751395

[43] Zhang X Ma N Lin Q Chen K Zheng F Wu J . Body roundness index and all-cause mortality among US adults. JAMA Netw Open. (2024) 7:e2415051. doi: 10.1001/jamanetworkopen.2024.15051, 38837158 PMC11154161

[44] Goran MI Ball GD Cruz ML. Obesity and risk of type 2 diabetes and cardiovascular disease in children and adolescents. J Clin Endocrinol Metab. (2003) 88:1417–27. doi: 10.1210/jc.2002-021442, 12679416

[45] Faty A Ferré P Commans S. The acute phase protein serum amyloid A induces lipolysis and inflammation in human adipocytes through distinct pathways. PLoS One. (2012) 7:e34031. doi: 10.1371/journal.pone.0034031, 22532826 PMC3331860

[46] Mirzababaei A Abaj F Khosravinia D Ghorbani M Valisoltani N Clark CCT . The mediatory effect of inflammatory markers on the association between a body shape index and body roundness index with cardiometabolic risk factor in overweight and obese women: a cross-sectional study. Front Nutr. (2023) 10:1178829. doi: 10.3389/fnut.2023.1178829, 37360300 PMC10288880

[47] Jialal I Jialal G Devaraj S Adams-Huet B. The effect of increasing tertiles of waist circumference on cardio-metabolic risk, adipokines and biomarkers of inflammation and oxidative stress in nascent metabolic syndrome. J Diabetes Complicat. (2018) 32:379–83. doi: 10.1016/j.jdiacomp.2018.01.008, 29478813

[48] Guo W Lu J Qin P Li X Zhu W Wu J . The triglyceride-glucose index is associated with the severity of hepatic steatosis and the presence of liver fibrosis in non-alcoholic fatty liver disease: a cross-sectional study in Chinese adults. Lipids Health Dis. (2020) 19:218. doi: 10.1186/s12944-020-01393-6, 33028338 PMC7541277

[49] Zou H Ma X Zhang F Xie Y. Comparison of the diagnostic performance of twelve noninvasive scores of metabolic dysfunction-associated fatty liver disease. Lipids Health Dis. (2023) 22:145. doi: 10.1186/s12944-023-01902-3, 37674196 PMC10481547

[50] Schuster S Cabrera D Arrese M Feldstein AE. Triggering and resolution of inflammation in NASH. Nat Rev Gastroenterol Hepatol. (2018) 15:349–64. doi: 10.1038/s41575-018-0009-6, 29740166

[51] Rowland M McGee A Broderick A Drumm B Connolly L Daly LE . Repeatability of transient elastography in children. Pediatr Res. (2020) 88:587–92. doi: 10.1038/s41390-020-0916-4, 32357363

[52] Pv Alves V Trout A Dewit M Mouzaki M Arce-Clachar AC Bramlage K. Clinical performance of transient elastography with comparison to quantitative magnetic resonance imaging, ultrasound, and biopsy in children and adolescents with known or suspected fatty liver disease. Childhood Obesity. (2023) 19:461–9. doi: 10.1089/chi.2022.0136 36269577

